# The impact of SARS-CoV-2 infection on the outcome of acute ischemic stroke—A retrospective cohort study

**DOI:** 10.1371/journal.pone.0282045

**Published:** 2023-03-02

**Authors:** Tímea Tünde Takács, Ádám József Berki, Péter Pál Böjti, Rita Stang, Pablo Antonio Fritz-Reunes, Luiz Schnekenberg, Timo Siepmann, Alexandra Pintér, Szabolcs Szatmári, Dániel Bereczki, Bence Gunda

**Affiliations:** 1 Semmelweis University, Department of Neurology, Budapest, Hungary; 2 Semmelweis University, “János Szentágothai” Doctoral School of Neurosciences, Budapest, Hungary; 3 National Institute of Mental Health, Neurology and Neurosurgery, Budapest, Hungary; 4 University Hospital Carl Gustav Carus, Department of Neurology, Dresden, Germany; 5 Division of Health Care Sciences, Center for Clinical Research and Management Education, Dresden International University, Dresden, Germany; 6 Semmelweis University, Department of Family Medicine, Budapest, Hungary; 7 MTA-SE Neuroepidemiological Research Group ELKH, Budapest, Hungary; 8 European Academy of Neurology, EANcore COVID-19 Task Force, Vienna, Austria; Stanford University School of Medicine, UNITED STATES

## Abstract

**Background:**

Acute ischemic stroke (AIS) is a common complication of severe acute respiratory syndrome coronavirus 2 (SARS‑CoV‑2) infection (COVID-19), but the prognosis of these patients is poorly understood.

**Purpose:**

To explore the impact of COVID-19 on neurological outcomes in AIS patients.

**Methods:**

A comparative retrospective cohort study was conducted in 32 consecutive AIS patients with and 51 without COVID-19 between the 1^st^ of March 2020 and 1^st^ of May 2021. The evaluation was based on a detailed chart review for demographic data, medical history, stroke severity, cranial and vessel imaging results, laboratory parameters, COVID-19 severity, hospitalization time, in-hospital mortality, and functional deficits at discharge (modified Rankin Scale, mRS).

**Results:**

COVID-19 AIS patients showed tendency to worse initial neurological deficit (NIHSS 9 (3–13) vs. 4 (2–10); p = 0.06), higher rate of large vessel occlusion (LVO; 13/32 vs. 14/51; p = 0.21), had prolonged hospitalization (19.4 ± 17.7 vs. 9.7 ± 7 days; p = 0.003), had lower chance of functional independence (mRS≤2) (12/32 vs. 32/51; p = 0.02) and showed higher in-hospital mortality (10/32 vs. 6/51; p = 0.02). In COVID-19 AIS patients, LVO was more common with COVID-19 pneumonia than without (55.6% vs. 23.1%; p = 0.139).

**Conclusion:**

COVID-19-related AIS carries a worse prognosis. COVID-19 with pneumonia seems to be associated with a higher rate of LVO.

## Introduction

Infection with severe acute respiratory syndrome coronavirus-2 (SARS-CoV-2) is associated with an increased risk for thromboembolic events. In fact, acute ischemic stroke (AIS) has been described as the most common cerebrovascular complication of Coronavirus disease 2019 (COVID-19) [1–5].

COVID-19 increases the risk of ischemic stroke via several pathophysiological mechanisms such as a proinflammatory immune response that triggers the coagulation cascade causing endothelial dysfunction resulting in a prothrombotic state with thrombotic micro-and macroangiopathy, downregulating the angiotensin-converting enzyme-2 receptors, and myocardial injury [3, 5–11]. Some initial studies have reported that COVID-19-related stroke affected many younger patients [12–15] with few or no risk factors, to be clinically more severe, to exhibit a greater number of vascular occlusions [16, 17], and to affect multiple brain areas [18, 19]. Yet, large cohort studies found and confirmed that the vast majority of AIS patients with concomitant COVID-19 infection are elderly with many cardiovascular risk factors, which are also risk factors for AIS [4, 20–22]. While the incidence of AIS in COVID-19 was initially thought to be as high as 3–6% [22, 23], subsequent studies agree that COVID-19 infection is associated with a lower stroke rate [21, 24–28] of about 1–1.5%. Further studies suggested that the risk of AIS increases with the clinical severity of COVID-19 [29–32]. Multiple cohort studies reported an overall high rate of discharge to a destination other than home and death in AIS patients with COVID-19 [2, 21, 22, 33].

Our research aimed to compare the differences between COVID-19 associated AIS patients and non-COVID-19 AIS patients regarding clinical, laboratory, imaging characteristics and outcomes at a Neurology ward also at the forefront of COVID-19 care.

## Material and methods

We retrospectively analyzed the data of all consecutive AIS patients with COVID-19 consulted in our hospital (N = 32) from 1^st^ of March 2020 to 1^st^ of May 2021 (first, second, and third COVID-19 wave in Hungary) and compared to the data of 51 consecutive non-COVID-19 AIS patients from our prospectively collected stroke registry during the second COVID-19 wave (October 2020) at the Department of Neurology, Semmelweis University, Budapest. We decided to choose October 2020 as the control time period because the second COVID-19 wave was already ongoing in Hungary, but our hospital was not yet involved in solely COVID-19 care. The study was approved by Semmelweis University Regional and Institutional Committee of Science and Research Ethics (No.: 201/2021).

Informed consent was not sought for the present study because of its observational and retrospective nature. All data were anonymized before analysis.

While admitting a few neurological patients, our department mostly took part in COVID-19 care. AIS was defined by presentation in the hospital with acute neurologic signs of stroke and confirmed ischemia on imaging (head Computed Tomography (CT) or Magnetic Resonance Imaging (MRI). In the COVID-19 AIS group patients with confirmed positive SARS-CoV-2 PCR test results within two weeks of stroke onset were selected, because in some cases at the time of the AIS event the COVID-19 test was yet negative, despite clinical symptoms, or they still might have been in the clinically prodromal phase of the infection. Patients were not tested routinely for COVID-19 on a daily basis, only in case of clinical suspicion, close contact of a diagnosed case or before transfer to another facility.

The collected data consisted of demographic data (sex, age), medical history (hypertension, diabetes, malignancy, smoking, ischemic cardiac disease, atrial fibrillation, previous stroke or transient ischemic attack, hyperlipemia, peripheral artery disease (PAD), chronic kidney- or lung disease); present stroke characteristics: admission National Institutes of Health Stroke Scale (NIHSS), etiology based on TOAST (Trial of ORG 10172 in acute stroke treatment) criteria, number and localization of brain infarcts, presence of large vessel occlusion (LVO), acute reperfusion therapy including intravenous thrombolysis (IVT) and endovascular therapy (EVT); laboratory tests (complete blood count, complete metabolic panel, C-reactive protein, and international normalized ratio); length of hospitalization, modified Rankin Scale (mRS, favorable functional outcome was defined as mRS≤2) at discharge, in-hospital mortality, and transfer to an intensive care unit (ICU) facility. Previous medical history was assessed based on prior medical documentation and current medications. Transfer to ICU in the non-COVID-19 group was calculated from 41 patients due to insufficient and unavailable follow-up data. In COVID-19 patients the presence of pneumonia was assessed based on chest CT or X-ray and COVID-19 severity based on WHO COVID-19 disease severity classification using four groups: mild, moderate, severe, and critical illness [34].

Statistical analysis was conducted between patient groups using either paired t-test, unpaired t-test, Mann-Whitney U test, univariable and multivariable logistic regression, linear regression, Chi-squared test, or Fisher’s exact test. The variables in the univariable logistic regression were selected based on the clinically most relevant cardiovascular risk factors (hypertension, diabetes mellitus, hyperlipemia, smoking, and other cardiovascular risk factors including history of chronic kidney disease, transient ischemic attack or stroke, ischemic heart disease, PAD, and atrial fibrillation) that can affect mortality in AIS patients. The factors with p<0.1 in univariable logistic regression were selected for multivariable logistic regression. Values are presented as mean ± standard deviation (SD) unless otherwise specified. For all analyses, an alpha value < 0.05 was considered significant. All analyses were performed using GraphPad Prism version 9.

## Results

Table 1 summarizes the results. There was no significant difference between COVID-19 AIS and non-COVID-19 AIS groups in age (70.1 ± 12.8 years vs. 70.6 ± 14.7 years; p = 0.68) and sex (male: 65.6% vs. 62.7%; p = 0.79). In both groups, there was a slight male predominance.

**Table 1:** Comparing characteristics of COVID-19 AIS with non-COVID-19 AIS patients.

Data	COVID-19 AIS	Non-COVID-19 AIS	Test	p-value
**Demographic data**				
Number of patients	32	51		
Age	70.1 (± 12.83)	70.7 (± 14.73)	Mann-Whitney U	0.68
Male sex (%)	21 (65.6%)	32 (62.7%)	Chi-squared	0.79
**Medical history**				
Diabetes (%)	11 (34.4%)	13 (25.5%)	Chi-squared	0.38
**Hypertension (%)**	**21 (65.6%)**	**44 (86.3%)**	**Chi-squared**	**0.02**
Hyperlipemia (%)	8 (25%)	18 (35%)	Chi-squared	0.33
Malignancy (%)	3 (9.4%)	7 (13.7%)	Fisher’s exact	0.73
Ischemic heart disease (%)	11 (34.4%)	14 (27.5%)	Chi-squared	0.50
Stroke/TIA (%)	6 (18.8%)	15 (29.4%)	Chi-squared	0.27
PAD (%)	5 (15.6%)	5 (10%)	Fisher’s exact	0.32
Chronic lung disease (%)	3 (9.4%)	3 (6%)	Fisher’s exact	0.67
Atrial fibrillation (%)	9 (28%)	9 (17.7%)	Chi-squared	0.25
Cardiac embolism (%)	11 (34.4%)	16 (31.4%)	Chi-squared	0.77
**Stroke characteristics**				
*Admission NIHSS (Median*, *IQR)*	*9 (3–13)*	*4 (2–10)*	*Mann-Whitney U*	*0*.*06*
LVO (%)	13 (40.6%)	14 (27.5%)	Chi-squared	0.21
Anterior LVO	12/13 (92.3%)	9/14 (64.2%)	Fisher’s exact	0.16
Posterior LVO	1/13 (7.7%)	4/14 (25.5%)	Fisher’s exact	0.32
Multilocular LVO	0/13	1/14 (7%)	Fisher’s exact	>0.99
Acute reperfusion therapy (IVT+EVT)	10 (31.3%)	12 (23.5%)	Chi-squared	0.43
IVT (%)	6 (19%)	6 (11.7%)	Chi-squared	0.37
EVT (%)	4 (12.5%)	6 (12%)	Fisher’s exact	>0.99
EVT/LVO (%)	4/13 (31%)	6/14 (43%)	Fisher’s exact	0.69
Door to needle time (minutes)	83 ± 35	54 ± 15	Unpaired t	0.17
Door to groin time (minutes)	378 ± 250	310 ± 221	Mann-Whitney U	0.33
**Laboratory findings**				
WBC 10^3^/μL	9.6 ± 4.3	9.73 ± 3.32	Mann-Whitney U	0.88
**Lymphocytes 10** ^**3**^ **/μL**	**1.54 ± 1.5**	**1.66 ± 0.7**	**Mann-Whitney U**	**0.04**
Thrombocyte 10^3^/μL	248.7 ± 86.1	250.2 ± 66.5	Unpaired t	0.92
Hemoglobin g/L	135.3 ± 22.2	142.1 ± 17.2	Mann-Whitney U	0.22
eGFR mL/min/1.73m^3^	69.05 ± 21.9	70.16 ± 21	Mann-Whitney U	0.55
**CRP mg/L**	**59.4 ± 68.4**	**21.89 ± 40.4**	**Mann-Whitney U**	**0.0012**
INR	1.09 ± 0.1	1.08 ± 0.2	Mann-Whitney U	0.16
**Outcome**				
**Hospitalization days**	**19.4 ± 17.7**	**9.7 ± 7**	**Mann-Whitney U**	**0.003**
*Discharge mRS (Median*, *IQR)*	*4 (1–6)*	*2 (1–4)*	*Mann-Whitney U*	*0*.*052*
**Favorable functional outcome (mRS≤2) (%)**	**12 (37.5%)**	**32 (62.8%)**	**Chi-squared**	**0.02**
**In-hospital mortality (%)**	**10 (31.3%)**	**6 (11.8%)**	**Chi-squared**	**0.02**
Transfer to ICU (%)	4 (12.5%)	1/41 (2.4%)	Fisher’s exact	0.16

Abbreviations: TIA: transient ischemic attack, PAD: peripheral artery disease, NIHSS: National Institutes of Health Stroke Scale, IQR: interquartile range, IVT: intravenous thrombolysis, EVT: endovascular therapy, LVO: large vessel occlusion, WBC: white blood cell count, eGFR: estimated glomerular filtration rate, CRP: C-reactive protein, INR: international normalized ratio, mRS: modified Rankin Scale. All values are presented as mean ± SD unless otherwise specified.

### Medical history

There was no significant difference in the history of diabetes, malignancy, ischemic heart disease, previous stroke or transient ischemic attack, hyperlipemia, PAD, chronic kidney or lung disease between the COVID-19 AIS and control group. In the COVID-19 AIS group the prevalence of hypertension was significantly lower (65.6% vs. 86.3%, p = 0.02).

### Stroke characteristics

At admission, the median (interquartile range, IQR) NIHSS of the COVID-19 AIS group showed a tendency to be higher (9 (3–13) vs. 4 (2–10); p = 0.06) but the difference was not statistically significant. On imaging (head CT or MRI) multiple vessel territorial involvement was similar in COVID-19 AIS and non-COVID-19 AIS groups (9.4% vs. 7.8%; p>0.05). We found a numerically higher LVO rate in the COVID-19 group (40.6% vs. 27.5%; p = 0.21). The proportion of anterior circulation LVO was higher in the COVID-19 AIS group (92.3% vs 64.2%).

**Fig 1:**
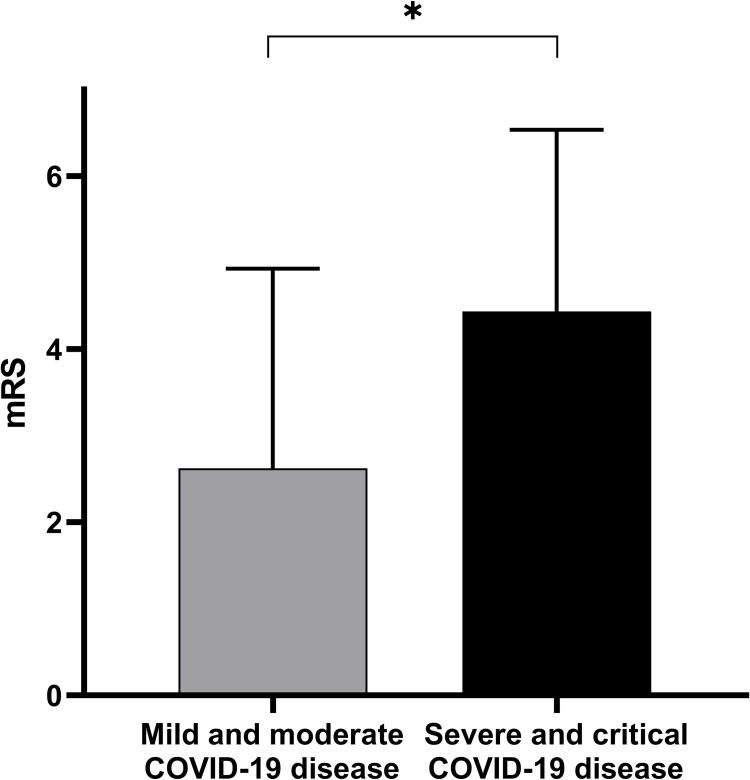
Relation of COVID-19 pneumonia and LVO in COVID-19 AIS patients. COVID-19 AIS patients with pneumonia more often had concomitant LVO (p = 0.139).

In the COVID-19 AIS group, LVO was more often seen in patients with COVID pneumonia than without (55.6% vs. 23.1%; p = 0.139, Fig 1).

Percentage of different stroke etiologies based on TOAST criteria in COVID-19 vs. non-COVID-19 AIS groups were the following: 14/32 (43.8%) vs. 18/51 (35.3%) undetermined, 11/32 (34.4%) vs. 16/51 (31.4%) cardioembolic, 1/32 (3.1%) vs. 7/51 (13.7%) small vessel disease, 6/32 (18.8%) vs. 6/51 (11.8%) large artery atherosclerosis, and 0/32 (0%) vs. 4/51 (7.9%) other determined causes. None of the differences were significant (p>0.05).

In our study, 31.3% of COVID-19 AIS patients underwent acute reperfusion therapy, out of which 6/32 (18.8%) had IVT and 4/32 (12.5%) EVT, no patient was eligible for both. In the control group, 23.5% were treated with acute reperfusion therapy, 6/51 (11.8%) received IVT, and 6/51 (11.8%) underwent EVT. One patient received both IVT and EVT. In the COVID-19 AIS group, the EVT/LVO ratio was 4/13 (30.8%), while in the control group 6/14 (42.9%), p = 0.69. The door to needle time in the COVID-19 AIS group was longer than in the non-COVID-19 AIS group (83 ± 35minutes vs. 54 ± 15 minutes; p = 0.17). Similarly, door to groin time was longer in the COVID-19 AIS group (378 ± 250 minutes vs. 310 ± 221 minutes. p = 0.33). None of the differences were significant.

### Laboratory findings

There was a significant difference in lymphocyte count (1.54 ± 1.5 103/μL vs. 1.66 ± 0.7 103/μl; p = 0.04) and C-reactive protein (CRP) levels (59.4 ± 68.4 mg/L vs. 21.9 ± 40.9 mg/L; p = 0.0012) between COVID-19 and non-COVID-19 AIS patients. No significant differences were seen in the other examined laboratory parameters.

### COVID-19 severity

In our COVID-19 cohort, 0/32 (0%) patients had mild, 16/32 patients (50%) had moderate, 12/32 (37.5%) had severe and 4/32 (12.5%) had critical COVID-19 severity based on the WHO COVID-19 disease severity classification.

**Fig 2:**
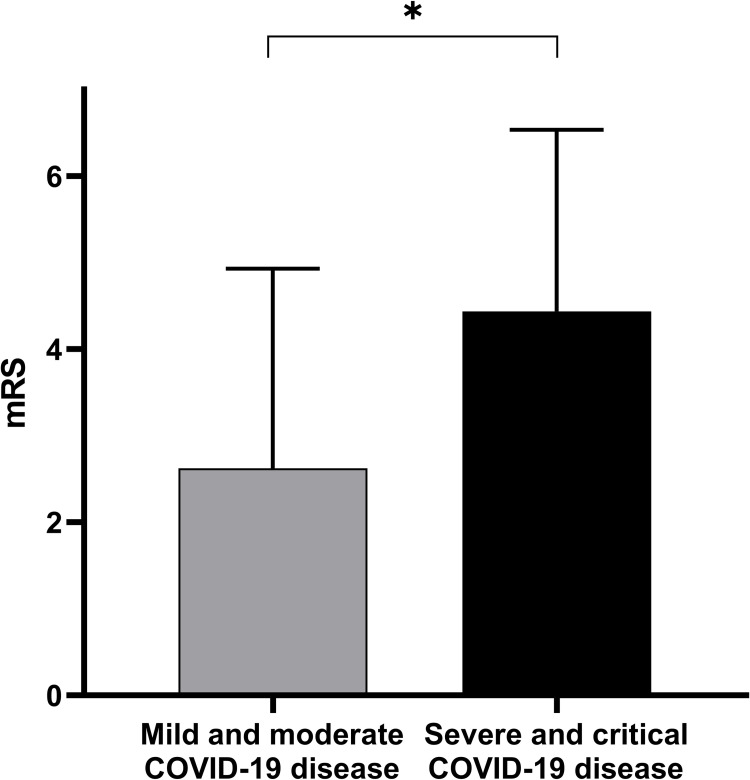
Association between COVID-19 severity and functional outcome. More severe COVID-19 infection resulted in worse functional outcomes (p = 0.01). Data are presented as mean ± SD, *p<0.05.

We assessed clinical characteristics in relation to COVID-19 severity dichotomized into mild-moderate vs. severe-critical groups (Table 2). There was no significant difference between NIHSS (median, IQR) at admission between the mild- moderate and severe-critical groups (9 (5–13.75) vs 6.50 (2.250–12.5); p = 0.30). The number of LVOs in the mild- moderate COVID-19 group was similar as in the severe- critical group (7/16 (43.75%) vs. 6/15 (40%); p = 0.83). Transfer to the ICU was only seen in the severe-critical COVID-19 group (0/16 (0%) vs. 4/16 (25%); p = 0.10). Duration of hospitalization was slightly longer in the severe-critical group (12.50 (7.500–18.75) days vs. 18.00 (6.750–33.00) days; p = 0.39). Severe- critical COVID-19 AIS patients showed moderately higher mortality (2/16 (12.5%) vs. 6/16 (37.5%), p = 0.43). We found a significant difference in the functional state at discharge between the two groups (2.6 ± 2.3 vs. mRS 4.4 ± 2.1; p = 0.014, Fig 2).

**Table 2:** Comparing clinical characteristics of mild-moderate vs. severe-critical COVID-19 AIS patients.

Data	Mild-moderate COVID-19	Severe-critical COVID-19	Test	p-value
Admission NIHSS (Median, IQR)	9 (5–13.7)	6.5 (2.2–12.5)	Mann-Whitney U	0.29
LVO	7/16 (43.7%)	6/15 (40%)	Chi-square	0.83
Transfer to ICU	0/16 (0%)	4/16 (25%)	Fisher’s exact	0.1
Hospitalization days (Median, IQR)	12.50 (7.5–18.7)	18.00 (6.7–33)	Mann-Whitney U	0.4
In-hospital mortality	2/16 (12.5%)	6/16 (37.5%)	Fisher’s exact	0.43
**Discharge mRS**	**2.6 ± 2.3**	**4.4 ± 2.1**	**Mann-Whitney U**	**0.01**

Abbreviations: NIHSS: National Institutes of Health Stroke Scale, IQR: interquartile range, LVO: large vessel occlusion, ICU: intensive care unit, mRS: modified Rankin Scale. All values are presented as mean ± SD unless otherwise specified.

### Hospitalization and outcome

**Fig 3:**
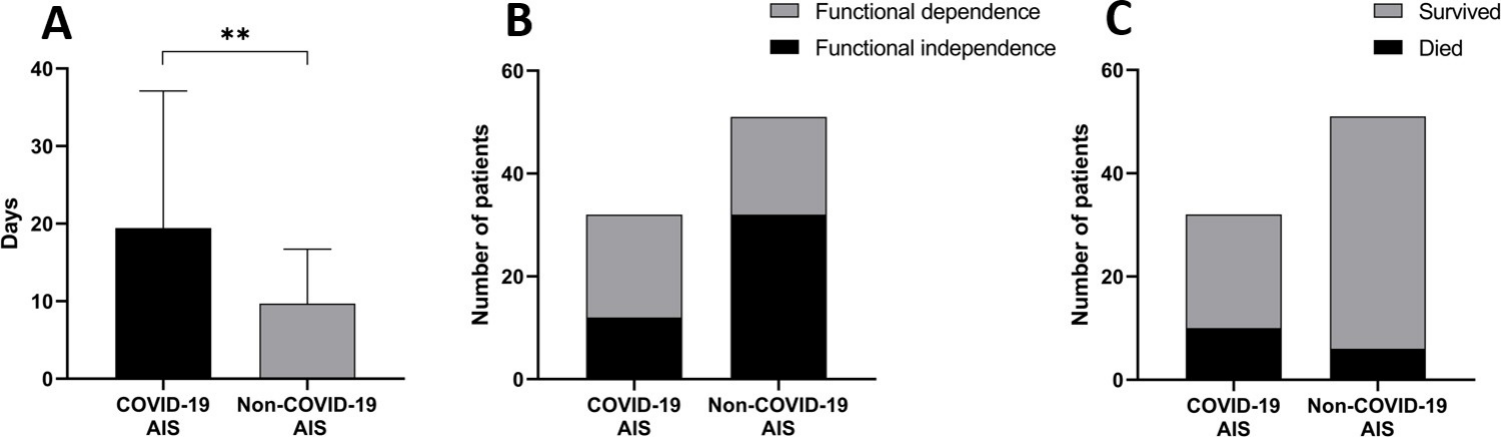
Summarizes the length of hospitalization and outcome result in COVID-19 AIS and non-COVID-19 AIS patients. (A). The average length of hospitalization. A significant difference between treatment duration can be observed. Data are presented as mean ± SD.**p<0.01. (B). Functional state at discharge. Non-COVID-19 AIS patients showed significantly more favorable functional outcomes (mRS ≤2) than COVID-19 patients; p = 0.02 (C). In-hospital mortality rates. There is significantly higher mortality in COVID-19 AIS patients; p = 0.02.

Length of hospitalization was prolonged in COVID-19 AIS patients when compared to the non-COVID-19 AIS group (19.4 ± 17.7 days vs. 9.7 ± 7 days; p = 0.003, Fig 3A). More COVID-19 infected AIS patients were admitted to ICU (4/32 (12.5%) vs. 1/41 (1.9%); p = 0.16). The median (IQR) discharge mRS was higher in the COVID-19 AIS group, but the difference was not statistically significant (4 (1–6) vs. 2 (1–4); p = 0.052). Yet, there were significantly fewer patients with a favorable functional outcome (mRS≤2) in COVID-19 AIS patients (12/32 vs. 32/51; p = 0.02, Fig 3B). In a subgroup analysis of anterior LVO patient’s functional outcome in the COVID-19 AIS group seemed less favorable compared to the non-COVID-19 AIS group (3.8 ± 2.5 vs. 2.7 ± 1.9, p = 0.22). In-hospital mortality was significantly higher (10/32 (31.3%) vs. 6/51 (11.8%); p = 0.02, Fig 3C) in the COVID-19 AIS population.

### Univariable and multivariable logistic regression analysis

With regard to all patients, in univariable logistic regression analysis there were significant associations between age and mortality (p = 0.04, odds ratio = 1.05, confidence interval = 1.005 to 1.102), between NIHSS and mortality (p = 0.0007, odds ratio = 1.17, confidence interval = 1.07 to 1.29), between CRP and mortality (p = 0.02, odds ratio = 1.010, confidence interval = 1.001 to 1.019) and between COVID-19 infection and mortality (p = 0.0339, odds ratio = 3.409, confidence interval = 1.122 to 11.18). There were no significant associations between diabetes mellitus and mortality (p = 0.151, odds ratio = 2.288, confidence interval = 0.72 to 7.116), between hypertension and mortality (p = 0.72, odds ratio = 0.792, confidence interval = 0.233 to 3.168), between hyperlipemia and mortality (p = 0.236, odds ratio = 0.441, confidence interval = 0.0943 to 1.54), between smoking and mortality (p = 0.200, odds ratio = 0.252, confidence interval = 0.013 to 1.421) and between other cardiovascular risk factors and mortality (p = 0.527, odds ratio = 1.435, confidence interval = 0.476 to 4.639). In multivariable logistic regression analysis NIHSS was found to be the only independent predictor of mortality (p = 0.004, odds ratio = 1.156, 95% confidence interval: 1.046–1.278), while age, CRP and COVID-19 infection were not.

We performed a sensitivity analysis restricted to patients in the NIHSS range of 5–15 generally considered as moderate stroke severity (Table 3). Hypertension remained significantly more common in non-COVID-19 AIS patients. There was a significant difference in CRP levels–significantly higher in the COVID-19 group, but not in lymphocyte count. Regarding outcome measures, only hospitalization days remained significantly longer in the COVID-19 AIS group. Discharge mRS and in-hospital mortality was still numerically worse in the COVID-19 group, although statistically not significant. These results show that in patients with comparable stroke severity, COVID-19 infection is liable for the longer hospitalization time and higher CRP, but not mortality. Also, in the multivariable and univariable logistic regression presented previously, NIHSS was found to be the only independent predictor of mortality, while COVID-19 infection was not.

**Table 3:** Comparing characteristics of COVID-19 AIS with non-COVID-19 AIS patients in NIHSS range 5–15.

Data	COVID-19 AIS	Non-COVID-19 AIS	Test	p-value
**Demographic data**				
Number of patients	15	21		
Age	70.1 (± 12.1)	75.7 (± 11.8)	Unpaired t	0.68
Male sex (%)	9 (60%)	13 (62%)	Chi-squared	0.90
**Medical history**				
Diabetes (%)	5 (33.3%)	6 (28.67%)	Fisher’s exact	0.99
**Hypertension (%)**	**9 (60%)**	**21 (100%)**	**Chi-squared**	**0.001**
Hyperlipemia (%)	2 (13%)	8 (38%)	Fisher’s exact	0.14
Malignancy (%)	1 (6.7%)	3 (14.2%)	Fisher’s exact	0.62
Ischemic heart disease (%)	4 (26.6%)	5 (23.8%)	Fisher’s exact	0.84
Stroke/TIA (%)	4 (26.7%)	7 (33.3%)	Fisher’s exact	0.72
PAD (%)	3 (20%)	2 (9.5%)	Fisher’s exact	0.62
Chronic lung disease (%)	2 (13.3%)	1 (4.7%)	Fisher’s exact	0.55
Atrial fibrillation (%)	5 (33.3%)	4 (19%)	Fisher’s exact	0.44
Cardiac embolism (%)	6 (40%)	10 (47.6%)	Chi-squared	0.65
**Stroke characteristics**				
Admission NIHSS (Median, IQR)	10 (8–11)	8 (6.5–12)	Unpaired t	0.83
LVO (%)	7 (46.7%)	8 (38%)	Chi-squared	0.60
Anterior LVO (%)	7/7 (100%)	5/8 (62.5%)	Fisher’s exact	0.2
Posterior LVO (%)	0/7	2/8 (22%)	Fisher’s exact	>0.99
Multilocular LVO (%)	0/7	1/8 (12.5%)	Fisher’s exact	>0.99
Acute reperfusion therapy (IVT+EVT) (%)	5 (33.3%)	6 (28.67%)	Fisher’s exact	>0.99
IVT (%)	2 (13.3%)	3 (14.2%)	Fisher’s exact	>0.99
EVT (%)	3 (20%)	3 (14.2%)	Fisher’s exact	>0.99
EVT/LVO (%)	3/7 (30.8%)	4/8 (50%)	Fisher’s exact	>0.99
**Laboratory findings**				
WBC 10^3^/μL	10.1 ± 5.5	10.9 ± 3.9	Unpaired t	0.88
Lymphocytes 10^3^/μL	1.7 ± 2.1	1.4 ± 0.6	Mann-Whitney U	0.69
Thrombocyte 10^3^/μL	276.1 ± 68.9	256.6 ± 60.5	Unpaired t	0.38
Hemoglobin g/L	130.6 ± 18.9	141.7 ± 16.7	Unpaired t	0.07
eGFR mL/min/1.73m^3^	70.6 ± 14.9	72.4 ± 17.1	Mann-Whitney U	0.27
**CRP mg/L**	**81.6 ± 74.1**	**30.6 ± 53.5**	**Mann-Whitney U**	**0.005**
INR	1.08 ± 0.1	1.07 ± 0.2	Mann-Whitney U	0.26
**Outcome**				
**Hospitalization days**	**18.4 ± 16.5**	**10.1 ± 7.9**	**Mann-Whitney U**	**0.04**
Discharge mRS (Median, IQR)	4 (1–6)	2 (2–4.25)	Mann-Whitney U	0.27
Favorable functional outcome (mRS≤2) (%)	3 (20%)	11 (52.3%)	Fisher’s exact	0.08
In-hospital mortality (%)	6 (40%)	3 (14.3%)	Fisher’s exact	0.12
Transfer to ICU (%)	2 (13.3%)	0/21 (0%)	Fisher’s exact	0.16

Abbreviations: TIA: transient ischemic attack, PAD: peripheral artery disease, NIHSS: National Institutes of Health Stroke Scale, IQR: interquartile range, IVT: intravenous thrombolysis, EVT: endovascular therapy, LVO: large vessel occlusion, WBC: white blood cell count, eGFR: estimated glomerular filtration rate, CRP: C-reactive protein, INR: international normalized ratio, mRS: modified Rankin Scale, ICU: intensive care unit. All values are presented as mean ± SD unless otherwise specified.

## Discussion

Our study showed that COVID-19 AIS patients had higher initial NIHSS, higher CRP, and lower lymphocyte count. Hospitalization time in the COVID-19 AIS group was longer, these patients had a higher rate of disability upon discharge and showed higher in-hospital mortality. We found an association between COVID-19-severity and discharge mRS and also a potential link between the presence of COVID-19 pneumonia and LVO.

There was no difference in age, similarly to data reported by *Harrison et al*. [20] and opposed to a higher average age in the non-COVID-19 AIS group reported by *Quresi et al*. [21]. There might be some younger COVID-19 AIS patients with a higher occurrence of LVO as initially reported [12–15], but after wide population reports, it is clear that both COVID-19 infection and AIS are more common in the elderly [20, 26, 35]. We found a slight male predominance in both groups similar to other studies [4, 25, 36], however, *Quresi et al*. found a women predominance in a large cohort study [21]. All stroke risk factors assessed in our study were reported to be more common in COVID-19 patients [4, 20, 21, 37], however not in other studies [21, 27]. In our data, only hypertension was more common in the non-COVID-19 group. The explanation can be, that in the non-COVID-19 group the proportion of small vessel disease etiology–usually associated with hypertension- is higher than in the COVID-19 group (7/51 (13.7%) vs. 1/32 (3.1%). There is an overall high prevalence of hypertension in Hungary (>60% after the age of 60) compared to other countries [38].Seeing that study results vary on average age, sex and comorbidities, it can be concluded that there is no clear correlation between these and COVID-19-related stroke, rather all factors are linked to cardiovascular vulnerability and increase the risk of suffering from both ischemic stroke and COVID-19 infection as well as an ischemic stroke as a complication of COVID-19.

In line with other reports [4, 18, 33, 36, 39–42], NIHSS upon admission was higher in COVID-19 AIS patients, median NIHSS being 9 in our study and 10 in *Ntaios et al*., a larger cohort study. Stroke severity varies in studies, but there is agreement on a higher stroke burden associated with COVID-19. Ischemic stroke etiology based on TOAST categories in our study is in accordance with available literature data: in COVID-19 patients there is a higher rate of large vessel disease, a similar percentage of cardioembolism, and a remarkably lower rate of small vessel disease [2, 36, 39, 43, 44].

There has been a noticeable drop in acute stroke hospitalization and acute reperfusion therapy numbers during the pandemic worldwide [33, 35, 45–51] as well as in Hungary [52, 53]. In the COVID-19 pandemic era, it is a struggle to harmonize the obligatory containment restrictions for COVID-19 and the “time is brain” concept of acute reperfusion. The extra precautions necessary for COVID-19 AIS patients inevitably slow down acute stroke management which can adversely affect the clinical outcome. Indeed, acute reperfusion therapy times showed a delay in the COVID-19 AIS group and can contribute to the worse clinical outcome [54, 55]. Almost a third of COVID-19 AIS patients underwent acute reperfusion therapy in our study, compared to approximately a quarter in the control group. This may be explained by the fact that COVID-19 AIS patients tend to have LVO-s more often and a higher stroke severity that is more alarming [42], thus more patients may have arrived within the therapeutic time windows. We have seen a higher percentage of acute reperfusion therapies in the COVID-19 AIS group compared to some studies (IVT: 18.75% vs. 19.7% *Ntagios et al*., 4.8% *Mathew et al*., 1*3*.*6% Shahjouei et al*. and EVT 11.8% vs. 12.1% *Ntaios et al*., 3.2% *Mathew et al*., 7.4% *Shahjouei et al*.). [4, 36, 44] A possible explanation for this might be, that during the second and third COVID-19 wave (from October 2020 to May 2021) our hospital canceled all acute neurological admissions and was solely committed to COVID-19 care, and only a few AIS patients were bought to the center by mistake, usually as thrombolysis candidates. The relatively higher EVT rate compared to a slightly lower than usual IVT rate (of about 26% [52]) might be explained by the delay in recognizing stroke signs due to at-home or in-hospital isolation and also by fear of hospitalization during the pandemic. The strict IVT time window of 4.5 hours without the additional multimodal perfusion imaging data (CT perfusion or MRI perfusion) unavailable in COVID-19 care had often passed, so IVT was contraindicated. The alarming factor of severe symptoms and the longer time window for mechanical thrombectomy resulted in more referrals to EVT, if applicable. The lower EVT/LVO ratio in the COVID-19 AIS group can be explained by the worse overall clinical state and prognosis of COVID-19 patients together with the unavailability of mechanical thrombectomy on-site, which meant that patients eligible for EVT were strictly selected. A higher rate of multiple territory infarcts in COVID-19 patients [18] was not confirmed in our study.

Similar to other studies [14, 16, 17, 44] we found a higher rate of LVO in COVID-19 vs. non-COVID-19 AIS-s (40.6% vs. 27.5%), although it was not significant. A novel finding in our study is that the higher rate of LVO was mainly driven by COVID-19 patients with pneumonia, as 55% of COVID-19 patients with pneumonia had LVO, while only 23% of patients without pneumonia presented with LVO. These results suggest that there might be a stronger relationship between COVID-19 pneumonia and LVO. It seems to be biologically plausible but requires further investigations. More severe COVID-19 infection regardless of presence of pneumonia showed no clear association with higher LVO rate and longer hospitalization, but might correlate with higher mortality rate according to our study.

Lower lymphocyte count [27, 41] and higher CRP [22, 39, 56] in COVID-19 AIS patients are similar to other studies and are associated with a poor prognosis of AIS in COVID-19 [41, 56, 57].

The worse prognosis of the COVID-19 AIS group, even with the small number of patients, is the most robust result in our study. The hospitalization period was two-fold longer in COVID-19 patients in our study, similar to the findings of *Mathew et al*. [2, 36]. Longer hospitalization was due to both COVID-19 associated infection with strict containment regulations, and to the overall worse clinical state of COVID-19 AIS patients. During hospitalization, more COVID-19 AIS patients needed to be transferred to ICU, but our data show a remarkably lower transfer rate than other cohort studies [4, 21, 25]. The explanation might be that COVID-19 patients admitted to our hospital usually had low-medium COVID-19 infection severity at admission, more severe cases were sent to Internal medicine, Pulmonology COVID-19 wards, or ICU. The worse functional state at discharge in the COVID-19 AIS group suggests that COVID-19 infection has a major role in determining the outcome in AIS patients [4, 58]. This is also supported by a higher rate of independent functional outcome in non-COVID-19 patients [4, 36]. In our study, 62.5% had mRS ≥3 in the COVID-19 AIS group, which is similar to 69.7% found by *Mathew et al*. [36]. Despite the higher proportion of the more benign anterior LVO in COVID-19 AIS, clinical outcome was still worse in this group. Comparing only anterior LVO patients, COVID-19 AIS was associated with a worse outcome, which also indicates that the simultaneous COVID-19 infection affects the overall clinical outcome [59–61].

In accordance with numerous studies, we found higher in-hospital mortality of about 30% in the COVID-19 AIS group, which is similar to large study results [2, 4, 18, 20–22, 33, 39, 41, 62]. The higher mortality of the control group compared to previous data from our hospital (11.8% vs. 7.5%) [63] can be explained by the stay-at-home regulations, fear of the pandemic, and the fact that mostly patients with severe neurological symptoms sought medical help, sometimes too late for acute reperfusion therapy [52]. We conducted an additional logistic regression analysis to find various factors associated with mortality in our patient population. We found that in univariate testing the presence of COVID-19 infection is a strong predictor of in-hospital mortality (odds ratio = 3.409), but not independent from age, NIHSS, and CRP. In one study COVID-19 infection was reported to be an independent risk factor for mortality [62]. In our study, although with a small sample size, baseline NIHSS was found to be the only independent predicting factor of mortality, while age, COVID-19 infection severity, and CRP were not. This finding highlights that the elderly, with more cerebrovascular risk factors, are prone to both COVID-19 infection and AIS alone, but also to ischemic stroke as a complication of COVID-19 infection.

In summary, our results suggest that AIS together with COVID-19 infection results in a more severe neurological deficit, worse functional outcome, and higher mortality than non-COVID-19 AIS. Severe COVID-19 infection associated with worse outcomes. This finding underlines the importance of a personalized multi-disciplinary approach to these vulnerable patients including neurological, pulmonological, and intensive care expertise.

## Limitation

The main limitation of our study is the small number of patients that resulted in apparently clinically meaningful differences not reaching statistical significance. Another important limitation is the retrospective nature of our research, therefore we could only assess laboratory tests and other available patient data, and discharge mRS was determined based on medical documentation, not patient interviews.

## Conclusion

In our single-center population of AIS patients, infection with SARS-CoV-2 was associated with more severe neurological deficits, higher mortality, prolonged hospitalization, and worse functional outcome. COVID-19 pneumonia seems to be associated with a higher rate of LVO. Clinical prognosis seems to further deteriorate with the severity of COVID-19 underscoring the potential value of multi-disciplinary care in this population at risk.

## Supporting information

S1 Data (PDF)
