# Supplementary material for: The impact of SARS-CoV-2 infection on the outcome of acute ischemic stroke—A retrospective cohort study
Source: PLoS One. 2023 Mar 2;18(3):e0282045. doi: 10.1371/journal.pone.0282045 (PMC9980769; doi:10.1371/journal.pone.0282045)
Supplement: S1 Data — (PDF) [file pone.0282045.s001.pdf]

Basic Data				Stroke characteristics								
patient nr	COVID/non COVI	age	male=0; female=	stroke_yes=1; no	TOAST	Cardioembolic(1=	NIHSS admission	Imaging: location	LVO_yes=1; 0=no	LVO_1=one; 2=m	IVT_yes=1; 0=no	EVT_yes=1; 0=no
1	COVID	62	0	1	5=undetermined	0	8	1	1	1	0	1
2	COVID	62	0	1	5=undetermined	0	4	1	0	0	0	0
3	COVID	61	0	1	2=cardioemb	1	3	2	1	1	0	0
4	COVID	95	1	1	2=cardioemb	1	11	1	0	0	0	0
5	COVID	65	1	1	5=undetermined	0	9	1	0	0	0	0
6	COVID	53	0	1	3=smallvessel	0	2	2	0	0	0	0
7	COVID	74	0	1	5=undetermined	0	2	1	0	0	0	0
8	COVID	81	1	1	5=undetermined	0	13	1	0	0	0	0
9	COVID	43	0	1	2=cardioemb	1	3	3	0	0	1	0
10	COVID	82	0	1	2=cardioemb	1	13	1	1	2	0	0
11	COVID	67	0	1	1=LargeArtAthero	0	1	2	0	0	0	0
12	COVID	68	0	1	5=undetermined	0	4	1	0	0	0	0
13	COVID	59	1	1	5=undetermined	0	1	1	0	0	0	0
14	COVID	53	0	1	2=cardioemb	1	10	1	1	1	0	1
15	COVID	73	0	1	5=undetermined	0	11	1	1	0	0	0
16	COVID	76	1	1	2=cardioemb	1	21	1	0	0	0	0
17	COVID	79	0	1	1=LargeArtAthero	0	3	1	1	1	1	0
18	COVID	94	1	1	2=cardioemb	1	19	1	0	0	0	0
19	COVID	65	0	1	5=undetermined	0	14	1	1	1	0	0
20	COVID	68	0	1	1=LargeArtAthero	0	7	1	1	1	1	0
21	COVID	88	1	1	5=undetermined	0	21	1	1	1	1	0
22	COVID	54	0	1	2=cardioemb	1	10	1	1	1	0	1
23	COVID	53	0	1	5=undetermined	0	9	1	1	1	0	1
24	COVID	76	1	1	2=cardioemb	1	21	1	1	1	0	0
25	COVID	71	0	1	1=LargeArtAthero	0	5	3	0	0	0	0
26	COVID	65	0	1	5=undetermined	0	3	1	0	0	0	0
27	COVID	82	1	1	1=LargeArtAthero	0	9	1	0	0	1	0
28	COVID	64	1	1	2=cardioemb	1	5	1	0	0	0	0
29	COVID	60	0	1	5=undetermined	0	18	1	1	2	1	0
30	COVID	88	0	1	1=LargeArtAthero	0	2	1	0	0	0	0
31	COVID	78	0	1	5=undetermined	0	28	3	n.a.		0	0
32	COVID	84	1	1	2=cardioemb	1	10	1	0	0	0	0
33	non-COVID	60	0	1	2=cardioemb	1	1	3	0	0	0	0
34	non-COVID	81	1	1	5=undetermined	0	7	1	0	0	0	0
35	non-COVID	43	0	1	6=ESUS	0	2	1	1	1	0	0
36	non-COVID	70	1	1	3=smallvessel	0	3	1	0	0	0	0
37	non-COVID	68	1	1	5=undetermined	0	6	2	0	0	0	0
38	non-COVID	75	0	1	1=LargeArtAthero	0	1	1	0	0	0	0
39	non-COVID	59	0	1	5=undetermined	0	0	1	0	0	0	0
40	non-COVID	60	0	1	6=ESUS	0	30	2	1	1	0	0
41	non-COVID	73	0	1	4=otherdetermine	0	14	2	1	1	0	1
42	non-COVID	61	0	1	2=cardioemb	1	7	2	1	0	0	0
43	non-COVID	90	0	1	5=undetermined	0	0	1	0	0	0	0
44	non-COVID	67	0	1	2=cardioemb	1	12	2	0	0	0	0
45	non-COVID	85	1	1	5=undetermined	0	4	2	0	0	0	0
46	non-COVID	58	0	1	3=smallvessel	0	2	1	0	0	0	0
47	non-COVID	67	0	1	5=undetermined	0	1	2	0	0	1	0

48	non-COVID	59	0	1	1=LargeArtAtherc	0	4	3	0	0	0	0
49	non-COVID	71	0	1	1=LargeArtAtherc	0	2	2	0	0	0	0
50	non-COVID	38	0	1	5=undetermined	0	4	2	1	2	0	1
51	non-COVID	92	0	1	2=cardioemb	1	3	1	0	0	0	0
52	non-COVID	89	1	1	2=cardioemb	1	4		0	0	1	0
53	non-COVID	77	1	1	5=undetermined	0	3	2	0	0	0	0
54	non-COVID	76	0	1	5=undetermined	0	4	1	0	0	0	0
55	non-COVID	46	1	1	5=undetermined	0	2	2	0	0	0	0
56	non-COVID	68	0	1	2=cardioemb	1	3	2	0	0	0	0
57	non-COVID	53	0	1	5=undetermined	0	1	1	0	0	0	0
58	non-COVID	48	0	1	3=smallvessel	0	3	1	0	0	0	0
59	non-COVID	85	0	1	5=undetermined	0	15	1	0	0	0	0
60	non-COVID	60	0	1	2=cardioemb	1	15	1	1	1	1	1
61	non-COVID	75	1	1	2=cardioemb	1	8	1	0	1	0	0
62	non-COVID	93	0	1	2=cardioemb	1	7	1	1	1	1	0
63	non-COVID	77	0	1	5=undetermined	0	11	2	0	0	0	0
64	non-COVID	81	0	1	2=cardioemb	1	10	1	1	0	0	1
65	non-COVID	83	1	1	1=LargeArtAtherc	0	16	1	1	1	1	0
66	non-COVID	44	0	1	3=smallvessel	0	8	1	0	0	0	0
67	non-COVID	90	1	1	2=cardioemb	1	11	1	0	0	0	0
68	non-COVID	66	1	1	2=cardioemb	1	1	1	0	0	0	0
69	non-COVID	62	0	1	1=LargeArtAtherc	0	5	1	1	1	0	1
70	non-COVID	80	0	1	2=cardioemb	1	8	1	0	0	0	0
71	non-COVID	87	1	1	2=cardioemb	1	6	1	0	0	1	0
72	non-COVID	79	0	1	3=smallvessel	0	6	1	1	1	0	0
73	non-COVID	86	1	1	3=smallvessel	0	9	2	0	0	0	0
74	non-COVID	56	0	1	4=otherdetermine	0	2	3	0	0	0	0
75	non-COVID	76	1	1	5=undetermined	0	6	1	0	0	0	0
76	non-COVID	77	1	1	2=cardioemb	1	22	1	1	1	0	1
77	non-COVID	50	0	1	5=undetermined	0	2	1	0	0	0	0
78	non-COVID	83	1	1	5=undetermined	0	4	2	0	0	0	0
79	non-COVID	85	0	1	5=undetermined	0	12	3	1	2	0	0
80	non-COVID	81	1	1	2=cardioemb	1	14	1	0	0	0	0
81	non-COVID	89	1	1	5=undetermined	0	20	1	1	1	0	0
82	non-COVID	45	0	1	3=smallvessel	0	2	1	0	0	0	0
83	non-COVID	79	1	1	1=LargeArtAtherc	0	2	1	0	0	0	0

	Previous Diagnoses											
WHO COVID seve	HT_yes=1; 0=no	DM_yes=1; no=0	malignancy_yes=	smoking_yes=1;	ischemic heart di	AF_ 1=yes; 0=no	stroke/TIA_yes=1	hiperlipid_yes=1;	PAD_yes=1; no=0	CKD_yes=1; no=0	CPD_yes=1; no=0	Anticoag 0=no; 1
3=hospital+O2 na	1	1	0	0	0	0	0	0	0	0	0	0
3=hospital+O2 na	1	1	0	0	0	0	0	1	0	0	0	0
2=hospital+no O2	1	0	0	1	0	1	0	1	1	0	0	0
3=hospital+O2 na	1	0	0	0	0	1	0	0	1	0	0	1
2=hospital+no O2	1	1	0	0	1	0	0	1	0	1	0	0
2=hospital+no O2	1	0	0	0	0	0	1	1	0	0	0	0
2=hospital+no O2	1	1	0	0	0	0	0	1	0	0	0	0
3=hospital+O2 na	1	0	1	0	0	0	1	0	0	0	1	0
2=hospital+no O2	0	0	0	0	0	0	0	0	0	0	1	0
2=hospital+no O2	0	0	0	0	1	1	0	0	0	0	0	0
2=hospital+no O2	0	0	0	0	1	0	0	0	0	0	0	0
2=hospital+no O2	0	1	0	1	0	0	0	0	0	0	0	0
2=hospital+no O2	0	0	0	0	0	0	0	0	0	0	0	0
2=hospital+no O2	0	0	0	0	0	0	0	0	0	0	0	0
2=hospital+no O2	1	1	0	0	0	0	0	0	0	0	0	0
2=hospital+no O2	1	0	0	0	1	1	0	0	0	0	0	0
2=hospital+no O2	1	1	0	0	0	1	0	0	0	0	0	0
2=hospital+no O2	1	0	1	0	1	1	1	0	0	0	0	0
3=hospital+O2 na	0	0	0	0	0	0	0	0	0	0	0	0
4= ICU	1	1	0	0	0	0	0	0	0	0	0	0
3=hospital+O2 na	1	1	0	0	1	0	0	1	0	1	0	0
2=hospital+no O2	0	0	0	0	0	0	0	0	0	0	0	0
4= ICU	0	0	0	0	0	0	0	0	0	0	0	0
2=hospital+no O2	1	0	0	0	1	1	0	0	0	0	0	0
3=hospital+O2 na	1	0	0	0	0	0	0	0	1	0	0	0
4= ICU	1	0	1	0	0	0	0	0	0	0	0	0
3=hospital+O2 na	0	0	0	0	0	0	1	0	1	0	0	0
3=hospital+O2 na	1	1	0	0	1	1	1	1	0	1	1	1
3=hospital+O2 na	1	1	0	0	1	0	0	1	1	0	0	0
3=hospital+O2 na	1	0	0	0	0	0	0	0	0	0	0	0
4= ICU	0	0	0	0	1	0	0	0	0	0	0	0
3=hospital+O2 na	1	0	0	0	1	1	1	0	0	0	0	0
0	0	0	0	0	0	0	1	1	0	0	0	0
0	1	0	0	0	0	0	0	1	0	0	0	0
1	0	0	1	0	0	0	0	0	0	0	0	0
1	1	0	0	1	0	0	0	0	0	0	0	0
0	1	0	0	1	0	0	1	0	0	0	0	0
0	1	0	0	0	0	0	0	0	0	0	0	0
0	1	0	0	1	1	0	0	1	1	1	0	0
0	1	0	0	1	0	0	0	0	0	0	0	0
1	1	0	0	0	0	0	0	0	0	0	0	0
0	1	0	0	1	0	0	0	0	1	0	0	0
0	0	0	0	0	0	0	1	0	0	0	0	0
0	1	1	1	0	1	0	0	1	0	0	0	0
	1	1	0	0	1	0	1	0	0	0	0	0
0	1	0	0	1	0	0	0	0	0		0	0
0	1	1	0	0	1	0	0	0	0	0	0	0

0	1	0	1	1	1	0	0	1	1	0	1	0
0	1	1	0	0	0	0	0	1	0	0	0	0
0	0	0	0	1	0	0	0	1	0	0	0	0
0	1	0	0	0	1	1	0	0	0	0	0	2
0	1	1	0	0	0	1	1	1	0	0	0	1
0	1	0	1	0	0	0	0	0	0	0	0	0
1	0	1	0	0	0	0	0	0	0	0	0	0
0	1	0	0	0	0	0	0	0	0	0	0	0
0	1	1	0	0	0	1	0	0	0	0	0	0
0	1	0	0	0	0	0	0	0	0	0	0	0
0	1	0	0	0	0	0	0	0	0	0	0	0
1	1	1	0	0	0	0	0	0	0	0	0	0
0	1	0	0	1	1	0	0	1	0	0	0	0
0	1	0	0	1	1	0	0	1	1	0	0	0
1	1	0	0	0	0	1	1	0	0	0	0	0
0	1	0	0	0	0	0	0	0	0	0	0	0
0	1	0	0	0	0	1	0	0	0	0	0	2
0	1	0	0	0	1	0	0	1	0	0	0	0
0	1	0	0	1	0	0	1	0	0	0	0	0
0	1	1	0	0	1	1	1	0	0	0	1	2
0	1	0	1	0	0	0	0	0	0	0	1	0
1	1	1	0	1	0	0	1	1	0	1	0	0
0	1	1	0	0	0	1	1	0	0	1	0	2
1	1	0	1	0	1	0	1	1	0	0	0	1
1	1	0	1	0	0	0	0	0	0	0	0	0
0	1	0	0	0	0	0	0	1	0	0	0	0
0	0	0	0	0	0	0	0	1	0	0	0	0
0	1	0	0	0	0	0	0	0	0	0	0	0
0	1	0	0	0	1	1	1	0	0	0	0	1
0	0	0	0	0	0	0	1	0	0	0	0	0
0	1	0	0	0	0	0	0	0	0	0	0	0
0	1	0	0	0	0	0	0	0	0	0	0	0
0	1	1	0	0	0	1	0	1	0	0	0	2
0	1	0	0	0	1	0	0	0	0	0	0	0
0	1	0	0	1	0	0	1	1	0	0	0	0
0	1	1	0	0	1	0	1	1	1	0	0	0

Lab Tests												
WBC	Ly	Thr	Hb	Htc	karbamid	kreatinin	eGFR	CRP	GOT	GPT	GGT	ALP
7.71	0.61	383	137	0.37	4.8	60	90	70.30	24	45	86	
10.12	1.29	418	135	0.36	3.6	51	90	124.2	94	96	73	88
9.84	2.36	289	142	0.41	6.5	85	84.3	6.3	23	28	36	67
3.1	1.34	303	104	0.32	5	74	59.2	46.2	13	4	18	69
12.79	1.44	320	127	0.38	10.5	132	36.4	16		13	22	57
4.02	0.63	207	144	0.44	7.3	108	67.3	8.6		36	81	81
8.19	1.8	177	156	0.44	6.4	90	71.8	1.5	14	10	16	66
4.54	9	324	137	0.39	13.7	88	53.3	88.7	48	12	17	43
11.84	1.18	194	153	0.42	8.5	76	90	2.1	24	43	22	72
16	1.33	329	125	0.36	4.8	65	86	118.4		21	25	
6	1.45	171	144	0.41	5.7	58	90	1	11	10	19	
7.61	2.08	261	122	0.38	3.1	59	90	9.2	38	71	41	65
7.81	1.74	319	152	0.42	92	5.6	81.1	10.3	27	56	59	69
8.29	1.66	362	135	0.39	3	66	79.9	1.9	27	17	13	96
10.76	1.18	300	178	0.54	10.3	59	85.6	6.8	79	60	581	356
7.16	1.21	148	150	0.43	5.7	69	85.7	6.4	23	22	54	60
8.39	3.2	225	146	0.43	9.3	114	35.6	10.1	42	51	94	202
15.2	1	125	133	0.4	5.3	108	61.4	16.2	44	28	30	
14.9	1.28	316	137	0.39	7.9	80	86.4	199.1	37	41	34	
4.63	0.38	123	151	0.41	16.1	121	34.3	188.9	61	16	22	
7.8	1.74	319	152	0.42	5.6	92	81	10.3	27	56	59	
9.78	0.7	231	136	0.37	8.9	=;	76.6	201.6	146	104	494	139
10.76	1.18	300	178	0.54	10.3	59	85.6	6.8	79	60	581	356
7.86	1.18	247	159	0.44	5.7	67	90	91.2	37	42	137	
10.46	0.67	271	83	0.24	56.2	476	10.2	21.3	22	23	83	96
3.7	0.5	155	94	0.3	2.7	62	79.8	24.7	51	12	20	
9.36	1.05	246	104	0.32	5.4	73	74.8	169.9	14	9	67	
7.03	1.26	147	133	0.36	12.9	145	43.3	1.7	48	98	104	
13.5	1.15	177	115	0.35	9.7	86	69.1	139.4	30	36	43	104
14.67	1.66	54	100	0.32	10.1	178	30.8	92.9	146	25	137	256
23.75	0.66	269	133	0.38	6.4	76	61.9	148	22	17	379	
6.7	2.68	282	144				73	1.4				
14.16	0.97	253	163				87.5	1.3				
6.15	1.35	184	135				90	46.7				
13.9	0.83	309	146				55	105.8				
9.98	1.21	343	140				90	5.7				
7.3	2.56	252	128				61.9	1.1				
9.94	1.99	448	136				86	20.8				
16.35	0.44	33	183				21.6	9.8				
19.68	2.01	224	142				71.4	230				
10.96	0.99	196	141				90	6.3				
6.85	2.16	208	155				60.6	0.6				
11.28	1.33	276	177				72.5	9.6				
7.01	1.8	208	116				37.9	3.6				
8.85	0.89	214	169				20.2	6.9				
7.46	1.46	269	151				90					

11.72	1.43	209	99	90	93.2
10.09	1.42	234	167	90	3.6
6.46	1.18	248	135	90	1.3
5.66	1.6	173	118	61.7	29.7
7.99	2.44	216	128	50.8	2.1
7.1	1.58	221	136	90	22.3
13.5	2.48	319	169	89	2.7
8.03	2.05	242	141.8	90	5
8.84	1.19	202	131	49.4	5.7
11.32	0.98	304	152	30	2.1
8.63	3.4	316	150	75.6	1
15.25	0.7	223	146	42.9	79.8
11.05	1.74	242	152	87.4	15.4
12.27	1	258	129	38	49.8
7.75	0.49	235	117	40	92.9
7.19	1.63	211	138	82.2	5.3
7.88	2	373	147	64	68
11.8	1.9	293	157	33	24
15.4	2.8	364	157	90	3.6
7.4	1.45	198	130	82	0.9
9.81	1.6	223	148	90	1.6
9.9	1.93	291	150	63	33.7
6.19	0.74	271	150	62.6	3.2
8.86	1	186	130	80.7	15.9
6.68	2.41	237	135	55.6	6.1
9.49	1.48	336	102	86.6	2.7
7.79	1.16	219	140	90	3.4
10.66	0.89	236	149	64.5	1
7.63	2.02	261	148		
7.44	2.19	304	144	90	4.8
5.99	1.84	154	118		
7.52	1.79	145	158	82.1	6
19.84	1.01	291	122	87.3	6.1
7.99	3.46	300	136	61.7	1.9
8.34	2.6	314	156	86.9	5.6
10.28	2.45	214	133	63.1	0.5

												Outc
INR	d-dimer	Trop	COVID pneumon	mCT(%)	echo_yes=1; no=	Holter_yes=1; no	AF_ 1=yes; 0=no	novumAF_yes=1;	ICU transfer_yes	hospital days	hospital mortalit	discharge_mRS
1.09	72		1	50	0	0	0	0	0	48	0	3
1.12			1	100	1	1	0	0	0	21	0	1
1	1.23		0	0	1	1	1	1	0	26	0	0
1.26	0.34		0	0	0	0	1	0	0	9	1	6
0.88			1	5	0	0	0	0	0	27	1	6
	0.34	7	0	0	0	0	0	0	0	15	0	1
			0	0	1	1	0	0	0	15	0	2
1.16	2.02		1	70	0	0	0	0	0	5	1	6
1.05	0.5		0	0	1	1	0	0	0	7	0	1
			1	75	0	0	1	0	0	9	1	6
			0	0	1	1	0	0	0	9	0	1
1.06	0.8		1	5	0	0	0	0	0	7	0	1
			0	0	0	0	0	0	0	1	0	1
1.01	1.3	4	0	10	1	1	0	1	0	7	0	0
1.05	1.07	6	1	50	1	1	0	0	0	20	0	3
1.26			0	0	0	0	1	0	0	57	0	5
1.18	1.28		1	150	0	0	1	0	0	12	0	5
1.05	3.8	64	0	0	0	0	1	0	0	13	0	5
1.29	>4,52	48	1	80	0	0	0	0	0	17	1	6
1.09	>4,45		1	150	1	1	0	0	1	15	0	0
1.23		672	1	100	0	0	0	0	0	24	1	6
1.01	1.3	4	1	20	1	1	0	1	0	10	0	0
0.91	2.67	8	1	180	0	0	0	0	1	6	0	6
1.26			0	0	0	0	1	0	0	15	0	5
1.12		16	0	0	0	0	0	0	0	70	0	3
1.05	>4,52	190	1	125	1	0	0	1	1	65	1	6
1.06	>4,31	38	1	10	1	1	0	0	0	36	0	5
1.09	0.53		1	10	0	0	1	0	0	12	0	3
0.97	1.62		1	50	0	0	0	0	0	4	1	6
1.01	>4,31	30	1	150	1	0	0	0	0	20	0	2
>7			1	180	0	0	0	0	1	1	1	6
1.16	2.8	52	0	0	0	0	1	0	0	19	1	6
1.14	0.32				1	1	0	1	0	1	0	1
					1	1	0	0	0	14	0	4
					0	0	0	0	0	6	0	1
0.96					1	1	0	0	0	12	0	3
					1	1	0	0	0	14	0	1
1.12					1	1	0	0	0	5	0	1
0.92					0	0	0	0	0	14	0	0
					0	0	0	0	0	3	1	6
1.02					0	0	0	0	0	26	1	6
	1.23				1	1	0	1	0	17	0	2
					0	0	0	0	0	1	0	1
0.96					1	1	0	1	0	15	0	2
					0	0	0	0	0	9	1	6
					0	0	0	0	0	7	0	1
1					1	0	0	0	0	7	0	0

			0	0	0	0	0	20	0	2
1.17			1	1	0	0	0	9	0	1
0.9			1	1	0	0	0	29	0	3
1.05			0	0	1	0	0	8	0	4
1.74			0	0	1	0	0	3	0	1
			0	1	0	0	0	8	0	1
			0	0	0	0	1	7	1	6
			0	1	0	0	0	9	0	2
0.98			0	1	1	0	0	7	0	1
1.06			0	0	0	0	0	6	0	0
1.04			1	1	0	0	0	6	0	1
			0	0	0	0	0	5	1	6
0.97			0	0	0	0	0	5	0	1
1.01			1	1	0	0	0	10	0	2
1.1			0	0	1	0	0	13	0	4
1			1	1	0	0	0	30	0	5
			0	0	1	0	0	5	0	2
1.09			0	0	0	0	0	2	0	5
1.05	0.77		1	1	0	0	0	8	0	2
1.02			0	0	1	0	0	3	1	6
1.11		7	0	1	0	1	0	10	0	0
1.06			1	1	0	0	0	7	0	2
1.7			0	0	1	0	0	4	0	2
1.12			0	0	0	0	0	10	0	3
			0	0	0	0	0	5	0	0
0.98		20	1	1	0	0	0	18	0	4
		3	0	0	0	0	0	1	0	1
			0	0	0	0	0	1	0	1
1.09			0	0	1	0	0	1	0	5
			0	0	0	0	0	1	0	1
			0	0	0	0	0	1	0	2
			0	0	0	0	0	1	0	4
1.03		28	0	0	1	0	0	1	0	5
			0	0	0	0	0	1	0	5
		4	0	0	0	0	0	1	0	1
			0	0	0	0	0	1	0	1

omes

1 month death_	3 months death_	1 month mRS	3 month mRS
0	4		
0			
0	0		
0	0	0	0
0	1	5	6
1		6	
0	0	5	
0	0		
1		6	
0	0	2	
0			
